# Isolation and Probiotic Functions of *Bacillus subtilis* and Its Inhibitory Effects on Colitis

**DOI:** 10.3390/biology14121786

**Published:** 2025-12-15

**Authors:** Ningning Guan, Chang Li, Wei Liu, Qiong Wu, Jiajia Zhu, Ting Gao, Hui Song, Rui Guo, Fangyan Yuan, Yongxiang Tian, Keli Yang, Danna Zhou

**Affiliations:** 1 Hubei Provincial Key Laboratory of Animal Pathogenic Microbiology, Institute of Animal Husbandry and Veterinary, Hubei Academy of Agricultural Sciences, Wuhan 430064, China; 2 College of Veterinary Medicine, Huazhong Agricultural University, Wuhan 430070, China; 3 Key Laboratory of Prevention and Control Agents for Animal Bacteriosis, Ministry of Agriculture and Rural Affairs, Wuhan 430064, China

**Keywords:** *Bacillus subtilis*, probiotic properties, DSS, colitis

## Abstract

*Bacillus subtilis*, as a probiotic feed additive, has been increasingly applied in livestock and poultry farming. In the present study, the environmental tolerance of a strain of *Bacillus subtilis*, isolated from a goat farm, was investigated. This article conducts a series of experiments on the obtained strains. The results demonstrated that the isolated strain exhibits strong tolerance to high temperatures, acidic and alkaline conditions, and high concentrations of bile salts. Furthermore, its self-aggregation rate exceeded 60% after 24 h. Whole-genome sequencing revealed that the genomes of the isolated strains were functionally annotated, identifying genes associated with amino acid metabolism, vitamin biosynthesis, and other metabolic pathways. Based on this genomic analysis, the present study further evaluated the anti-inflammatory effects of a *Bacillus subtilis* strain isolated in a murine model of colitis induced by dextran sulfate sodium (DSS). The analyses revealed that the DSS-treated group exhibited significantly reduced expression of intestinal tight junction proteins ZO-1 and Occludin, along with elevated expression of pro-inflammatory cytokines, compared to the PBS control group. Following oral administration of 1 × 10^8^ CFU/mL *Bacillus subtilis* isolated strain suspension, the DSS-treated mice showed increased expression of ZO-1 and Occludin and decreased levels of pro-inflammatory cytokines. These results indicate that the isolated strain of *Bacillus subtilis* has a protective effect against colitis and demonstrates probiotic potential.

## Simple Summary

*Bacillus subtilis*, recognized as a strain with potential probiotic properties, exhibits strong tolerance to acidic, alkaline, and high-temperature environments, and is widely utilized as a feed additive in livestock and poultry production to promote healthy growth. In recent years, intestinal inflammation has received increasing attention, and emerging evidence indicates that *Bacillus subtilis* may exert inhibitory effects on gut inflammatory responses. In the present study, stress resistance assays were performed on a clinically isolated strain of *Bacillus subtilis*, confirming its robust resilience under adverse conditions. Furthermore, in a murine model of colitis, administration of *Bacillus subtilis* was associated with the significant attenuation of intestinal inflammatory symptoms in the experimental group relative to the control group, indicating a potential therapeutic role for this strain in the amelioration of colitis.

## 1. Introduction

In recent years, research into the replacement of antibiotics with probiotics has gained increasing attention. Moreover, the beneficial effects of using probiotics as feed additives to enhance the growth and development of livestock and poultry, as well as to prevent and treat diseases, have been increasingly recognized [1]. The identification and application of high-quality probiotics are therefore of great importance. According to the International Scientific Association for Probiotics and Prebiotics (ISAPP), the consumption of adequate amounts of probiotics can positively contribute to the host’s health and development [2]. Among the various probiotic strains, *Bacillus subtilis* has attracted significant interest in the field of functional food research. Compared with other probiotics, *Bacillus subtilis* exhibits strong resistance to environmental stressors, such as acidity, alkalinity, and high temperatures, enabling it to survive and exert probiotic effects in the gastrointestinal tract [3]. Furthermore, *Bacillus subtilis* demonstrates superior stability during the processing and storage of food and pharmaceutical products. Numerous studies have indicated that this strain possesses promising probiotic potential, offering antimicrobial properties, anti-inflammatory effects, the ability to maintain intestinal microbial balance, and improvements in growth performance [4].

Due to changes in dietary habits and lifestyle, inflammatory bowel disease (IBD), which was once prevalent in North America and Europe, has seen a significant rise in newly industrialized countries such as those in Asia and Africa [5]. The clinical manifestations primarily include diarrhea, unformed stools with blood, decreased appetite, and weight loss [6]. IBD is classified as a group of autoimmune diseases characterized by chronic gastrointestinal inflammation resulting from dysfunctions in the innate immune system. This condition involves multiple factors, including infectious agents and genetic predisposition [7]. IBD is further categorized into Crohn’s disease and ulcerative colitis. In recent years, nearly all pharmacological treatments for IBD have focused on modulating the immune response [8]. Nevertheless, the widespread clinical use of these treatments is largely constrained by their considerable expenses and high rates of disease recurrence [9]. In parallel, patients with IBD frequently exhibit marked changes in both the structure and functional behavior of their gut microbiota. Such disruptions in microbial equilibrium can lead to intestinal dysbiosis, potentially increasing the risk of additional medical complications [10]. Today, there is increasing utilization of probiotic product, aimed at regulating intestinal microbiota as a means to prevent and treat IBD [11].

*Bacillus subtilis*, as a probiotic with potential therapeutic applications, has been widely studied for its efficacy in disease treatment [12]. *Bacillus subtilis* has been demonstrated to enhance intestinal barrier function and improve immune responses in broilers affected by necrotic enteritis [13]. It has significantly mitigated the toxic effects of deoxynivalenol-contaminated Fusarium enol residue on intestinal flora and tissues in piglet diets [14], and reduced the expression of pro-inflammatory factors in peripheral blood mononuclear cells, indicating preclinical anti-inflammatory properties [15]. Compared with traditional probiotics like *Lactobacillus* and *Bifidobacterium*, *Bacillus subtilis* displays enhanced tolerance to environmental challenges. This resilience is largely attributed to its ability to generate transient spores, which allow it to survive the low pH of the gastrointestinal environment and resist bile salt exposure, thereby supporting successful gut colonization and sustained physiological activity. Thanks to its durable spore-forming nature, *Bacillus subtilis* outperforms *Lactobacillus* and *Bifidobacterium* in maintaining viability under harsh processing and prolonged storage conditions, making it a promising probiotic choice for animal feed applications in livestock and poultry farming [16].

In the present study, the probiotic potential of an isolated strain of *Bacillus subtilis* was preliminarily analyzed. Its ability to tolerate harsh environmental conditions, including acidic and alkaline conditions, high salinity, high temperature, and gastrointestinal fluids, as well as its self-aggregation capacity, was evaluated. The administration of DSS was used to induce colitis in mice. Subsequently, the isolated *Bacillus subtilis* was administered via gavage to assess its therapeutic potential in the context of IBD in mice. The inhibitory effects of *Bacillus subtilis* on IBD were evaluated through monitoring the body weight change rate, measuring the expression levels of pro-inflammatory cytokines, conducting histopathological analysis of the colonic mucosa, and examining the immunohistochemical (IHC) expression of intestinal tight junction proteins.

## 2. Materials and Methods

### 2.1. Animals, Strains, and Reagents

*Bacillus subtilis* was isolated from a goat farm in Tongcheng, Hubei, China. C57 female mice, 4 weeks old, were purchased from Hubei Provincial Center for Disease Control and Prevention. The animal study was approved by the Ethics Committee of the Institute of Animal Husbandry and Veterinary, Hubei Academy of Agricultural Sciences (Wuhan, China; regulation ID: SCXK-2024-0016).

Tryptone soy agar (TSA), tryptone soy broth (TSB), Dextran sulfate sodium salt (DSS), 2 × TSINGKE^®^ Master qPCR Mix (SYBR Green I), HiScript III All-in-one RT SuperMix Perfect for qPCR and Nuclease-free Water were purchased from Qingdao Haibo Biologics (Qingdao, China), Beijing Puxitang Biotechnology Co., Ltd. (Beijing, China) and Vazyme (Nanjing, China), respectively.

### 2.2. Bacterial Isolation, Staining, and Microscopic Observation

A total of 36 oral and nasal swab samples were collected from goats using sterile instruments and promptly transported on ice to the laboratory for subsequent analysis. The samples were inoculated onto TSA plates using the streak plate method with a disposable sterile inoculation loop, followed by incubation in an inverted position at 37 °C for 24 h. Single colonies were picked from the plates and subcultured to achieve pure isolates, followed by the selection of one isolated colony for Gram staining, and examine the morphology of the bacteria strain under an oil immersion microscope. And the strain was inoculated into 10 mL of LB broth medium and incubated at a constant temperature of 37 °C for 24 h, followed by storage at −80 °C in LB medium supplemented with 20% (*v*/*v*) sterile glycerol for later experiments.

### 2.3. Molecular Identification of Bacillus subtilis Isolates

The bacterial strains were inoculated into TSB medium, and the turbid bacterial cultures were harvested after incubation at 37 °C and 170 rpm for 24 h. 16S rRNA sequence of each isolate was amplified using universal primers 27F (5′-AGAGTTTGATCCTGGCTCAG-3′) and 1492R (5′-GGTTACCTTGTTACGACTT-3′) (Reference strains Accession: AB523721.1 and AB523722.1). The PCR amplification was performed in a BIO-RAD thermal cycler with the following program: an initial denaturation step at 94 °C for 5 min, followed by 35 cycles consisting of 1 min at 95 °C for denaturation, 1 min at 58 °C for annealing, and 2 min at 72 °C for extension, with a final extension step at 72 °C for 5 min. PCR products were visualized by gel electrophoresis, purified by MiniBEST Agarose Gel DNA Extraction Kit (TaKaRa, Beijing, China) and were sent to Beijing Qingke Biotechnology Co., Ltd. (Beijing, China) for sequencing and further analysis. The 16S rRNA sequences were compared with reference strains in the GenBank database using BLAST 2.17.0, and a phylogenetic tree was constructed using MEGA 11 software for evolutionary analysis.

### 2.4. Determination of Growth Curve

The bacterial suspension was adjusted to a concentration of 1 × 10^8^ CFU/mL and then diluted 100-fold to obtain a final density of 1 × 10^6^ CFU/mL. Subsequently, 100 μL of the diluted suspension was transferred into a 96-well plate. The OD_600_ absorbance was measured at 4 h intervals over a 48 h period to construct the bacterial growth curve.

### 2.5. Assessment of Environmental Tolerance

#### 2.5.1. Tolerance of Acid

Firstly, the pH of PBS buffer was adjusted to 2.0, 3.0, 4.0, 8.0, 9.0, and 10.0 using hydrochloric acid and sodium hydroxide. Subsequently, a bacterial suspension by inoculating 1 mL of 1 × 10^8^ CFU/mL *Bacillus subtilis* culture was prepared into 9 mL PBS buffer at each designated pH value. A PBS buffer with a pH of 7.0 served as the control group. A 10-fold serial dilution of the bacterial suspension was carried out following incubation at 37 °C for 3 h. An appropriate dilution factor was then selected, and 100 μL of the diluted bacterial solution was plated using glass beads for colony counting. The survival rate was calculated as follows: Survival Rate (%) = (Number of *Bacillus subtilis* colonies in the test group/Number of *Bacillus subtilis* colonies in the control group) × 100%.

#### 2.5.2. Tolerance of High-Temperature

A bacterial solution of *Bacillus subtilis* at a concentration of 1 × 10^8^ CFU/mL was subjected to heat treatment at 37, 50, 60, and 80 °C in a water bath for 30 min. Following treatment, the solution was serially diluted tenfold, and the survival rate was calculated using the sample treated at 37 °C as the control [17]. The calculation method was consistent with that described in Section 2.5.1.

#### 2.5.3. Bile Salt Tolerance Assay

The TSB culture medium was prepared using pig bile salt (bile oxgall ≥ 65%) to achieve bile salt concentrations of 0.1%, 0.2%, 0.3%, 0.4%, and 0.5%. Subsequently, 1 mL of *Bacillus subtilis* bacterial suspension was inoculated into each of the TSB media containing bile salts. The TSB medium without bile salt served as control group. Following incubation at 37 °C for 3 h, an appropriate dilution gradient was selected, and 100 μL of the bacterial suspension was plated using glass beads for colony counting. The survival rate was then calculated. The calculation method followed the procedure described in Section 2.5.1.

#### 2.5.4. Tolerance to Artificial Gastrointestinal Fluid

A 1 mL aliquot of *Bacillus subtilis* bacterial suspension at a concentration of 1 × 10^8^ CFU/mL was inoculated into 9 mL of prepared artificial gastric juice (Pepsin 0.1 g/L, pH 3.5) and incubated at 37 °C for 3 h. The bacterial suspension was serially diluted tenfold every hour. An appropriate dilution gradient was selected, and 100 μL of the diluted suspension was spread onto agar plates using glass beads for colony counting, and the survival rate was calculated accordingly. Subsequently, 1 mL of the artificial gastric juice mixture was transferred into 9 mL of prepared artificial intestinal fluid (Trypsin 0.1 g/L, pH 6.8) and further incubated at 37 °C for 4 h, with tenfold serial dilutions performed hourly. Again, 100 μL of the appropriately diluted bacterial suspension was used for plate counting with glass beads. Sterile normal saline (pH 7.0) was used as the control for survival rate calculations [18], and the calculation method followed the procedure described in Section 2.5.1.

#### 2.5.5. Self-Aggregation Detection

The *Bacillus subtilis* bacterial suspension was centrifuged at 8000–10,000 rpm for 10 min. The supernatant was discarded, and the bacterial pellet was resuspended in PBS buffer to achieve a final concentration of 1 × 10^8^ CFU/mL. Subsequently, the suspension was incubated at 37 °C. The OD_600_ absorbance of the supernatant was measured at 0, 4, 8, 16, 20, and 24 h [19]. The self-aggregation experiment was performed in triplicate, and the data were averaged to ensure reliability. Self-aggregation was calculated using the following formula: Self-aggregation (%) = (1 − A_t_/A_0_) × 100, where A_t_ represents the absorbance at a specified time point and A_0_ denotes the absorbance at 0 h.

### 2.6. Whole Genome Sequencing Analysis

The whole genome sequencing and assembly of *Bacillus subtilis* were conducted by Beijing Qingke Biotechnology Co., Ltd. Genomic DNA was extracted from *Bacillus subtilis* and used for library construction, starting with 1 μg of total DNA. The DNA was sheared to fragments of approximately 300–500 bp using a Covaris M220 ultrasonicator (Covaris, Woburn, MA, USA), followed by end-repair, A-tailing, and adapter ligation (TruSeq™ Nano DNA Sample Prep Kit, Illumina, San Diego, CA, USA). The resulting library was amplified via PCR for 8 cycles, and the target fragments were purified using 2% Certified Low Range Ultra Agarose gel electrophoresis. The target DNA band was quantified using TBS380 (Picogreen, TurnerBioSystems, Sunnyvale, CA, USA), and subsequently loaded onto the sequencing machine according to the calculated data ratio. Bridge PCR amplification was then conducted on a cBot solid-phase carrier to generate DNA clusters. Using a second-generation sequencing platform, 2 × 150 bp paired-end sequencing was carried out, followed by the standard protocol of third-generation sequencing. Genomic DNA was fragmented into 8–10 kb fragments using the G-tubes method. The ends of the fragments were repaired and cyclized to form single-stranded-like structures. The single-stranded ends were then ligated to double-stranded adapters to generate a dumbbell-shaped (“horse ring”) structure. The single-stranded loop of the library was annealed and bound to polymerase immobilized at the bottom of zero-mode waveguides on the sequencing chip. Once the binding process was completed, the sequencing reaction was initiated on the instrument.

### 2.7. Development of Animal Models and DSS-Induced Experiment

Thirty-two mice were randomly assigned to four groups (*n* = 8 per group) according to body weight (BW): the blank control group (PBS), the model group (DSS), the intervention treatment group (DSS + BS), and the intervention control group (BS). The experimental procedures were as follows: (1) The PBS group was maintained on a standard diet for 14 days with ad libitum access to sterile drinking water. Additionally, 0.1 mL/10 g BW of PBS buffer was administered daily via gavage. (2) The DSS group was fed a standard diet for 14 days with ad libitum access to sterile drinking water. From day 8 to day 14, sterile water was replaced with a 3.5% DSS solution, and 0.1 mL/10 g BW of PBS buffer was administered daily via gavage. (3) The DSS + BS group was maintained on a standard diet for 14 days with ad libitum access to sterile drinking water. For the first 7 days, 0.1 mL/10 g BW of *Bacillus subtilis* bacterial suspension (1 mL, 1 × 10^8^ CFU/mL) was administered daily via gavage. From day 8 to day 14, sterile water was replaced with a 3.5% DSS solution, and the same bacterial suspension was continued daily via gavage. (4) The BS group was maintained on a standard diet with ad libitum access to sterile drinking water. Additionally, 0.1 mL/10 g BW of *Bacillus subtilis* bacterial suspension (1 mL, 1 × 10^8^ CFU/mL) was administered daily via gavage for 14 days. Throughout the experiment, the standard diet was feed at a rate of 12% of body weight, the room temperature was maintained at 23 °C to 25 °C, and the health status and clinical symptoms of the animals were monitored daily.

#### 2.7.1. Collection of Specimens

After the final dose, the mice were fasted for 24 h with access to sterile water only. Subsequently, the animals were euthanized using the cervical dislocation method. Following disinfection, the colon was carefully dissected and excised. The colon length was measured, and tissue samples were subsequently fixed in 4% paraformaldehyde solution and stored at −80 °C for further experimental analysis.

#### 2.7.2. Assessment of Disease Activity Index (DAI)

The DAI was utilized to evaluate the severity of colitis in mice. Daily observations were conducted to monitor changes in body weight, fecal consistency, and the presence of fecal blood, as previously described in the literature [20]. The specific scoring criteria are presented in Table 1.

#### 2.7.3. Colon Pathological Section

After resecting a segment of colon tissue, the specimen was fixed in a 4% paraformaldehyde solution, embedded in paraffin, and sectioned using a series of graded alcohol and xylene solutions. Histopathological changes in the colon were then examined under a microscope after hematoxylin and eosin (HE) staining.

#### 2.7.4. Colon Immunohistochemistry

According to Paul [21], the tissue sections were deparaffinized and rehydrated using alcohol and xylene, followed by antigen retrieval through acidic microwave treatment with citrate buffer (pH 6.0). Subsequently, the sections were incubated at room temperature to block nonspecific binding. The primary antibody, secondary antibody, and DAB chromogenic solution were then applied, and the sections were examined under a microscope. When a positive signal was observed in the tissue, the chromogenic reaction was terminated by rinsing with water and the sections were washed in water for 5 min.

### 2.8. Quantitative Real-Time PCR Analysis of the Expression of Inflammatory Factors

#### 2.8.1. Primers Design

The sequences were obtained from the NCBI database and the β-actin gene was selected in the internal reference.

The sequences of TNF-α, IL-1β, and IL-6 were obtained from the NCBI database, respectively, and the β-actin gene was selected as the internal reference. The primers were designed using Primer Premier 5.0 (Primer Biosoft International, Palo Alto, CA, USA) The primers were synthesized by Beijing Qingke Biotechnology Co., Ltd., and the primer sequences were shown in Table 2.

#### 2.8.2. RNA Isolation and Quantitative Real-Time PCR (qRT-PCR)

According to the manufacturer’s instruction of the RNA extraction kit (RC202-01, Vazyme), total RNA was extracted from the colon tissues, followed by reverse transcription into cDNA using the cDNA synthesis kit (R211-01, Vazyme).

The qRT-PCR was performed using TSINGKE^®^ Master qPCR Mix following the manufacturer’s instructions (TSE201, Tsingke, Beijing, China). The dissolution curve was acquired using the instrument’s default program. The housekeeping gene β-actin was amplified to normalize relative gene expression levels.

After amplification was completed, the melting curve was analyzed using the program provided by the PCR instrument (Tsingke) to determine the average Ct value of three replicate wells for each sample. The relative expression level of the target gene in each sample was calculated using the 2^−ΔΔCt^ method. The calculation formula is as follows: ΔCt = Ct (target gene) − Ct (internal reference gene), and ΔΔCt = ΔCt (test group) − ΔCt (control group).

### 2.9. Statistical Analysis

GraphPad Prism 8.0.2 software (GraphPad Software, USA) was used to analyze the experimental data and generate graphical representations. Data from each group were presented as mean ± standard deviation (SD). All experiments were repeated at least three times, and a *p*-value of less than 0.05 was considered statistically significant.

## 3. Results

### 3.1. Morphological and Molecular Identification of the Isolated Strain

**Figure 1:**
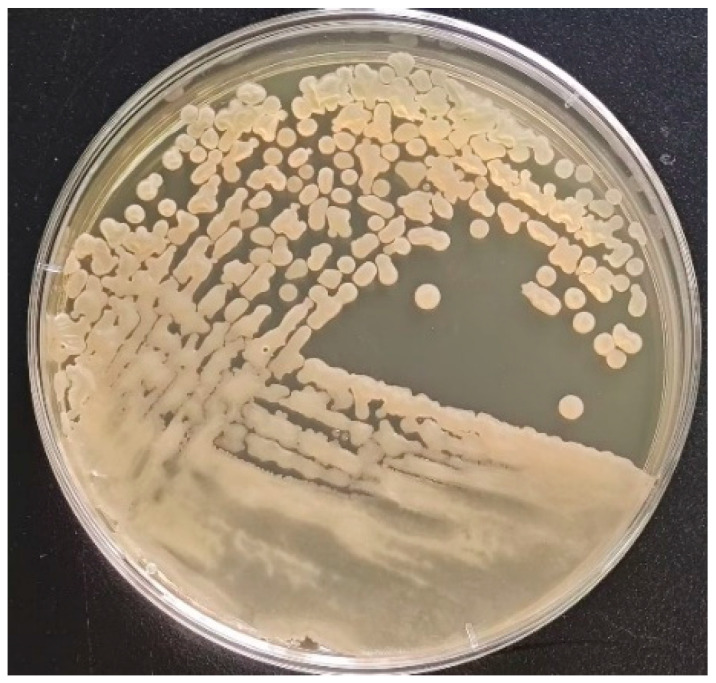
Colony morphology of the isolated strain on TSA plate. The colony surface is rough and opaque, appearing slightly yellow, with a diameter ranging from 1 to 2 mm, well-defined edges, and a centrally elevated morphology.

**Figure 2:**
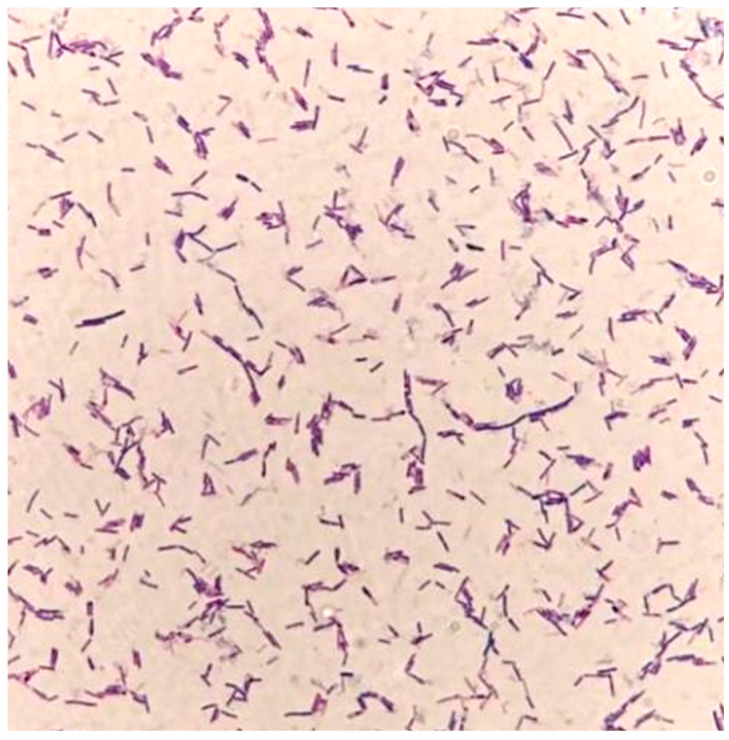
Gram staining of the isolated strain (100× magnification).

**Figure 3:**
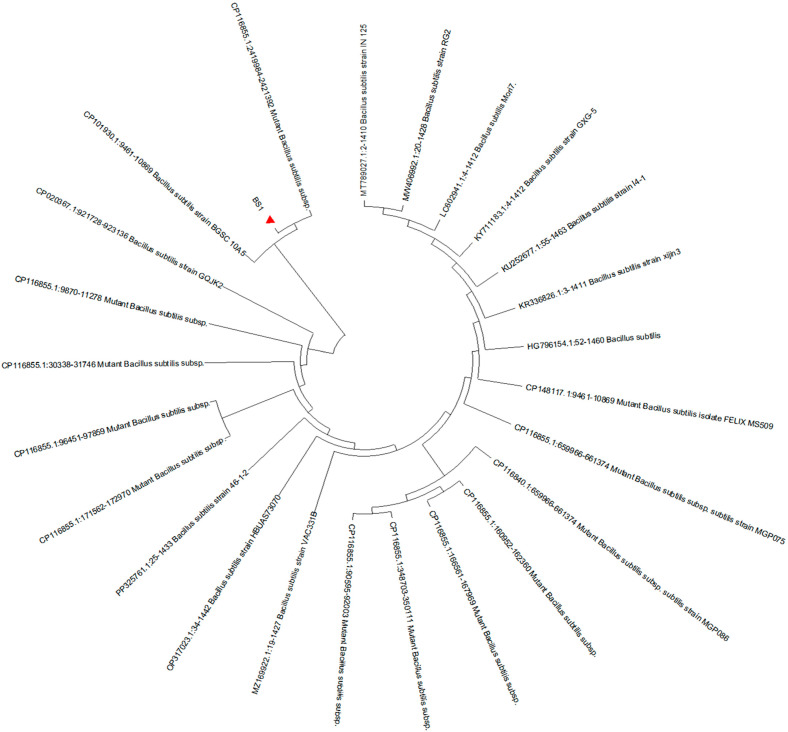
Neighbor-joining phylogenetic tree based on 16S rRNA sequences of isolated *Bacillus subtilis* strain. BS1 is the *Bacillus subtilis* strain in the present study.

In the present study, the isolated strain was obtained from the goats. On the TSA medium, a large, single colony displaying an off-white, opaque appearance with ruffled margins is observed (Figure 1). Microscopic examination reveals that the isolated strain appears as rod-shaped, Gram-positive bacteria, containing centrally or terminally located oval spores (Figure 2). Molecular analysis and BLAST searches based on the 16S rRNA sequence revealed that the isolated strain belonged to the genus Bacillus. Furthermore, sequence comparison of the 16S rRNA gene showed high similarity to *Bacillus subtilis*. Phylogenetic analysis of the 16S rRNA sequences indicated that the isolated strain clusters closely with *Bacillus subtilis* strain MGP075 (CP116855.1) on the phylogenetic tree (Figure 3). Based on the morphological characteristics and 16S rRNA analysis, the isolated strain was identified as *Bacillus subtilis.*

### 3.2. The Growth Curve and Stress Resistance of the Bacillus subtilis Isolated

**Figure 4:**
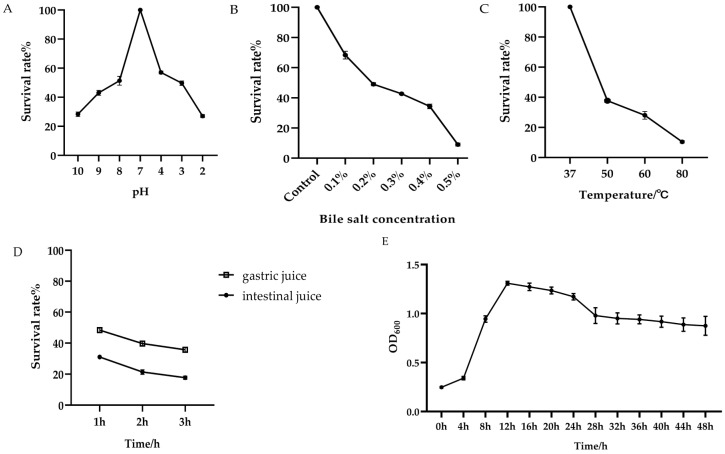
The growth curve and tolerance of the *Bacillus subtilis* to adverse environmental conditions. (**A**) Survival rate of the *Bacillus subtilis* under different pH conditions. (**B**) Survival rate of the *Bacillus subtilis* under varying bile salt concentrations. (**C**) Survival rate of the *Bacillus subtilis* at different temperatures. (**D**) Survival rate of the *Bacillus subtilis* in artificial gastric juice and artificial intestinal fluid. (**E**) The growth curve of the isolated strain of *Bacillus subtilis*.

As shown in Figure 4E, the isolated strain of *Bacillus subtilis* enters a phase of rapid growth between 4 and 12 h. During this period, bacterial growth and metabolic activity are most vigorous, which makes it an ideal time for subsequent experiments.

After incubation of the *Bacillus subtilis* in environments with pH levels ranging from 2.0 to 4.0 for 3 h, the survival rate ranged between 27% and 57%. When exposed to alkaline conditions with pH levels between 8.0 and 10.0 for the same duration, the survival rate ranged from 28% to 51% (Figure 4A). Salt tolerance experiments demonstrated that at a bile salt concentration of 0.1%, the survival rate reached 68%, whereas at 0.5%, the survival rate dropped significantly to 9% (Figure 4B). Following a 3 h incubation with artificial gastric juice, the survival rate decreased from 48% to 35%. After a similar incubation period with artificial intestinal fluid, the survival rate remained at 17.7% (Figure 4C). Notably, the *Bacillus subtilis* exhibited heat resistance, maintaining a survival rate of 10% even after exposure to 80 °C (Figure 4D). These findings indicate that the isolated strain of *Bacillus subtilis* possesses a certain degree of tolerance to adverse environmental conditions.

### 3.3. Stress Resistance in the Bacillus subtilis Isolated

**Figure 5:**
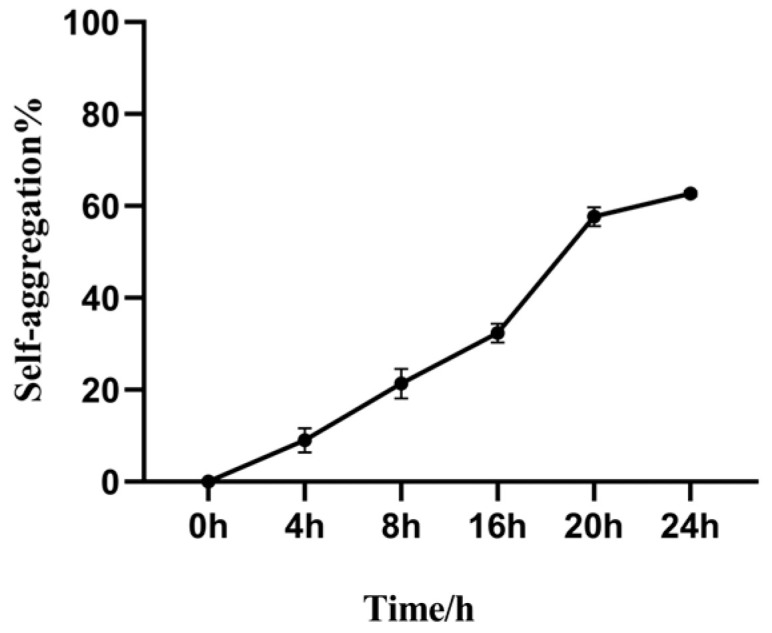
Self-aggregation capability of the *Bacillus subtilis*.

The self-aggregation ability is a critical factor in the functionality of the *Bacillus subtilis* within the animal intestine, as it facilitates the establishment of a sufficient bacterial density [22]. As shown in Figure 5, the *Bacillus subtilis* exhibits strong self-aggregation properties. Following incubation at 37 °C for 24 h, the self-aggregation rate was observed to reach 63%.

### 3.4. Whole Genome Sequencing of the Isolated Bacillus subtilis Strain

#### 3.4.1. Data Collection

**Figure 6:**
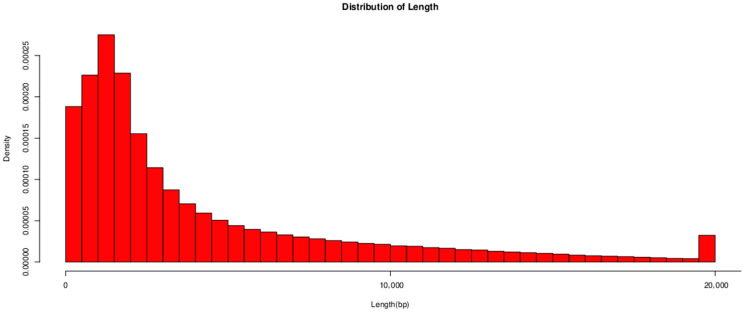
Statistical chart illustrating the length distribution of isolated *Bacillus subtilis*.

The quality control statistics for *Bacillus subtilis* are presented in Table 3. A total of 603,722 reads were generated, comprising 25,415,378,888 bases. The longest read length achieved was 69,930 bp, with N50 and N90 values of 8460 bp and 1830 bp, respectively. As illustrated in Figure 6, the read lengths and data volume were analyzed and found to be predominantly distributed within 20,000 bp. NCBI login number is: PRJNA1307218.

#### 3.4.2. Basic Genomic Characteristics of the Isolated *Bacillus subtilis*

**Figure 7:**
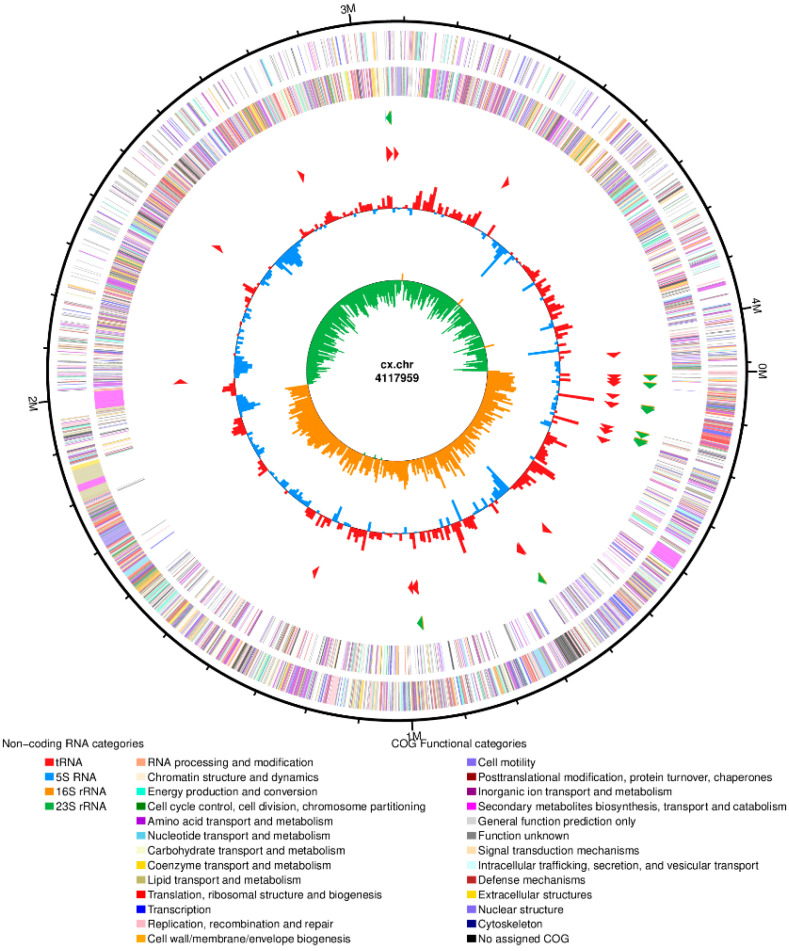
Genome map of the isolated strain of *Bacillus subtilis*.

After sequencing and assembly, as shown in Figure 7, the genome-wide overview of isolated strain *Bacillus subtilis* showed a ring chromosome with a total assembly length of 4,117,959 bp and consisted of a circular DNA structure. A total of 4276 protein-coding genes were identified, with a combined length of 366,822 base pairs and an average gene length of 857 base pairs. Additionally, the non-coding gene prediction results indicated that *Bacillus subtilis* strain contains 86 tRNA genes and 30 rRNA genes, including 10 copies each of 5S rRNA, 16S rRNA, and 23S rRNA. The prediction results for coding and non-coding genes are summarized in Table 4 and Table 5, respectively.

The outermost circle of the circle diagram is the identification of the size of the genome; the second and third circles are CDS on the positive and negative strands, and different colors represent the functional classification of different COGs of CDS; the fourth circle is rRNA and tRNA; the fifth circle is GC content, and the red part outward indicates that the GC content in this region is higher than the average GC content of the whole genome. The higher the peak, the greater the difference between the average GC content and the blue part in the inner direction indicates that the GC content in this region is lower than the average GC content of the whole genome. The higher the peak, the greater the difference between the average GC content; the innermost circle is GC skew value.

#### 3.4.3. COG Functional Classification Analysis

**Figure 8:**
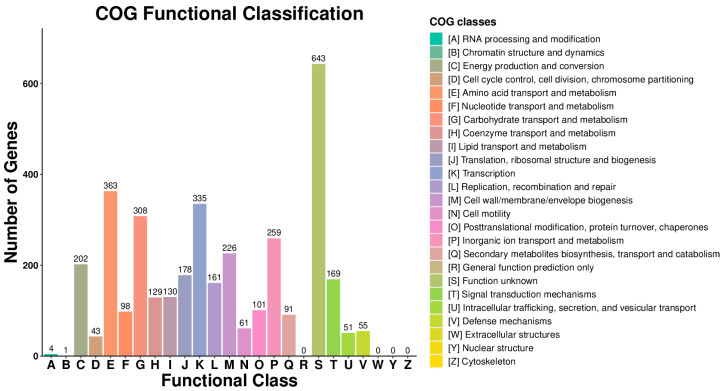
Statistical analysis of COG functional classification of *Bacillus subtilis* genomic proteins.

A COG database was constructed by comparing bacterial protein sequences, thereby facilitating functional annotation, classification, and evolutionary analysis of the predicted proteins in *Bacillus subtilis*. According to the results, excluding proteins of unknown function, the largest functional category was amino acid transport and metabolism, followed by transcription, carbohydrate transport and metabolism, inorganic ion transport and metabolism, and energy production and conversion (Figure 8).

#### 3.4.4. KEGG Pathway Analysis

**Figure 9:**
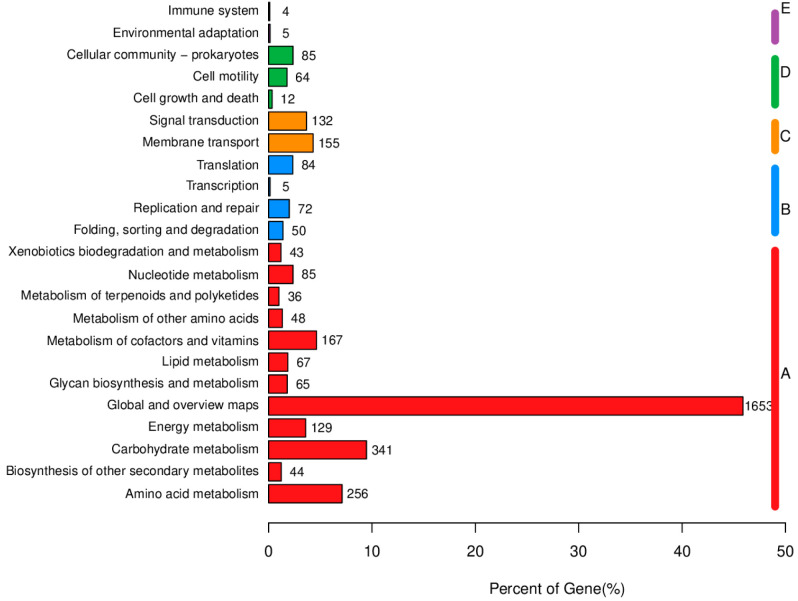
Functional classification results of KEGG annotation.

KEGG analysis results indicate that the majority of genes involved in metabolic processes are primarily associated with global and overview maps, carbohydrate metabolism, amino acid metabolism, energy metabolism, as well as cofactor and vitamin metabolism. With regard to genetic information processing, the predominant genes are primarily associated with replication, translation, and DNA repair, among other essential functions. In the context of environmental information processing, most genes are associated with membrane trafficking and signal transduction pathways. Regarding cellular processes, the majority of genes are related to cell communities—specifically in prokaryotes—along with cell motility, and cell growth and death. In terms of biological system functions, most genes are associated with environmental adaptation and immune system development (Figure 9).

#### 3.4.5. GO Database Annotations

**Figure 10:**
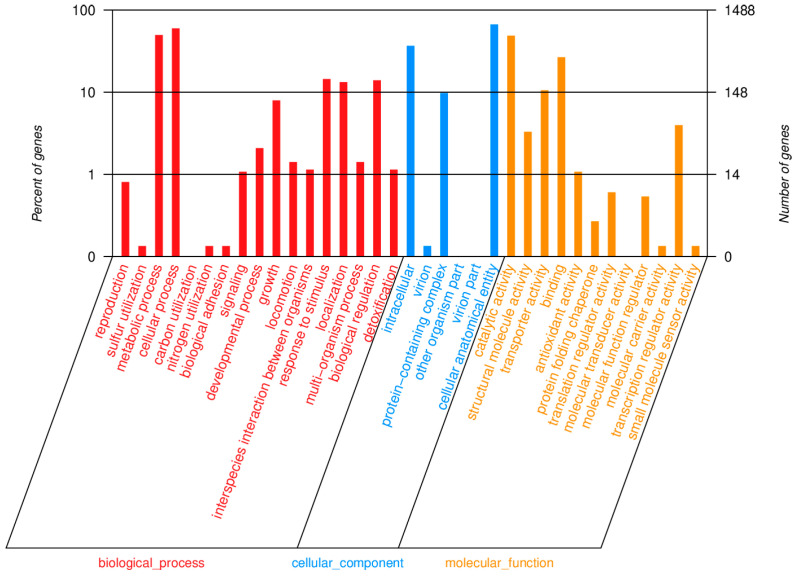
Functional classification results of GO annotation.

In the isolated strain of *Bacillus subtilis*, 1488 genes were annotated in the GO database, and these were mainly divided into biological processes (red), cellular components (blue) and molecular functions (orange). With respect to biological processes, the majority of genes in isolated strain are associated with cellular processes, metabolic activities, response to stimuli, localization, biological regulation, and growth. Regarding cell components, most genes are related to cell anatomical entities, intracellular components, and protein-containing complexes. In terms of molecular functions, the predominant activities include catalytic activity, adhesion, transporter activity, and transcriptional regulatory activity (Figure 10).

#### 3.4.6. CAZy Database Annotations

**Figure 11:**
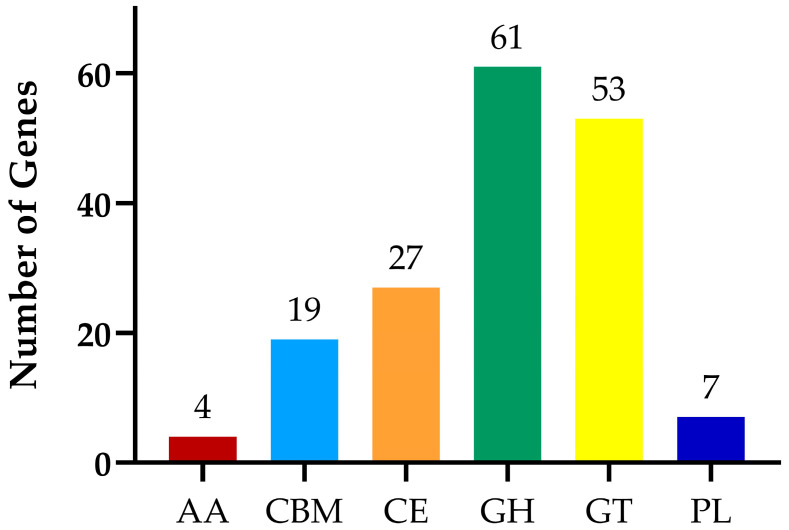
Distribution chart of carbohydrase.

The CAZy database annotation results are presented in Figure 11. The analysis reveals that a total of six functional categories were annotated, including glycoside hydrolases (GH), glycosyl transferases (GT), and carbohydrate esterases (CE), carbohydratebinding modules (CBM), polysaccharide lyases (PL), and auxiliary activities (AA). The number of genes involved in the annotated functions are 61, 53, 27, 19, 7, and 4, respectively.

#### 3.4.7. Antibiotic Resistance Gene Annotation

**Figure 12:**
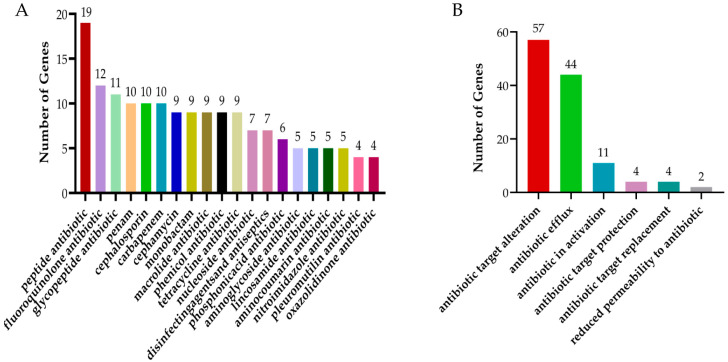
The results of statistical analysis of CARD. (**A**) CARD drug classification statistics. (**B**) Statistics of CARD action mechanism.

The statistical analysis of CARD revealed that the isolated *Bacillus subtilis* strain carry 19 genes conferring resistance to peptide antibiotics. This was followed by fluoroquinolones, glycopeptide antibiotics, carbapenems, and cephalosporins, which accounted for 12, 11, 10, and 10 resistance genes, respectively (Figure 12A). In terms of the mechanisms of antibiotic resistance, the most prevalent mechanism was antibiotic target modification, with a total of 57 associated genes, followed by antibiotic efflux (44 genes), antibiotic inactivation (11 genes), and antibiotic target protection (4 genes) (Figure 12B).

#### 3.4.8. Prediction of Protein Signal Peptides

**Figure 13:**
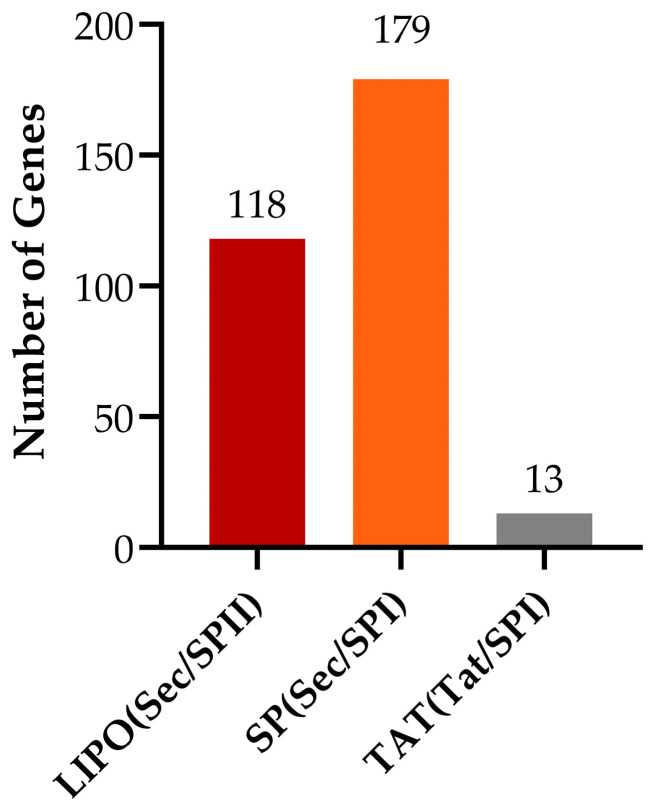
Statistical analysis of signal peptide types.

Signal peptides are short amino acid sequences that direct the translocation of newly synthesized proteins into the secretory pathway. Sequence analysis reveals that *Bacillus subtilis* possesses 118 standard secretory signal peptide genes transported via the Sec pathway and cleaved by signal peptidase I (Lep), 179 lipoprotein signal peptide genes also transported through the Sec pathway but cleaved by signal peptidase II (Lsp), and 13 Tat signal peptide genes transported via the Tat pathway and subsequently cleaved by signal peptidase I (Lep) (Figure 13).

### 3.5. The Effect of the Bacillus subtilis in the Alleviation of Colitis

#### 3.5.1. The Effect of *Bacillus subtilis* on Weight and DAI

**Figure 14:**
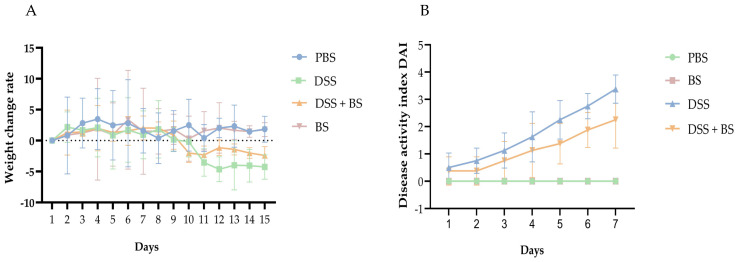
The weight change rate and the DAI rating of mice. (**A**) the weight change rate of mice. (**B**) the DAI rating of mice.

The main clinical manifestations of DSS-induced colitis in mice include bloody stools and weight loss, with weight changes serving as a reliable indicator of the animals’ response to colitis. As illustrated in Figure 14A, the weight change rate of all four groups of mice remained positive during the first seven days, indicating continuous weight gain. Starting on day 8, the DSS group and the DSS + BS group were administered DSS solution. From day 10 onward, the weight change rate in these two groups became consistently negative, indicating a sustained weight loss until the end of the experimental period. Notably, the weight loss in the DSS + BS group was slower compared to that in the DSS group. In contrast, the mice in the PBS group and the BS group experienced substantial weight gain throughout the study.

**Figure 15:**
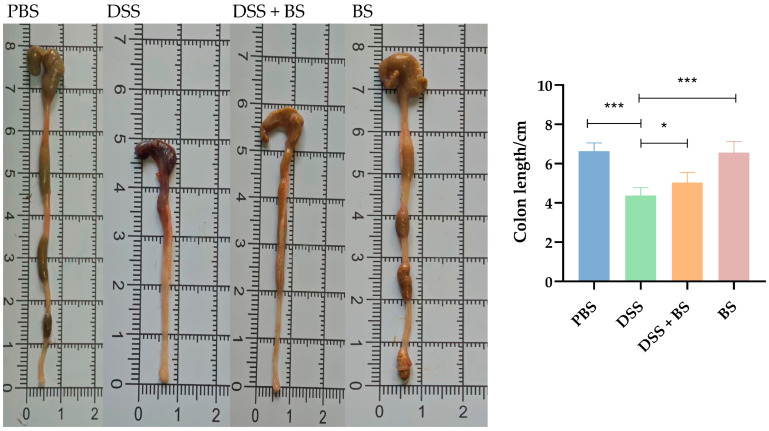
The colonal length of mice. The results are presented as mean ± SEM. The difference is not statistically significant (ns). * *p* < 0.05, indicates a significant difference, and *** *p* <0.001 indicates an extremely significant difference (*n* = 8).

To evaluate the severity of IBD in mice, the DAI score was applied, and the colon length was measured after dissection, as shown in Figure 14B and Figure 15. Following the administration of DSS in drinking water, the symptoms in the DSS group, including bloody stool and weight loss, progressively worsened over time. Compared with the PBS group and BS group, the colon length in the DSS group was significantly shorter. In contrast, mice treated with *Bacillus subtilis* exhibited longer colons and lower DAI scores, indicating a reduction in disease severity. These results indicated that the administration of *Bacillus subtilis* can partially alleviate the symptoms of experimental colitis.

#### 3.5.2. The Effect of *Bacillus subtilis* on Pathological Changes in Colonic Histology

**Figure 16:**
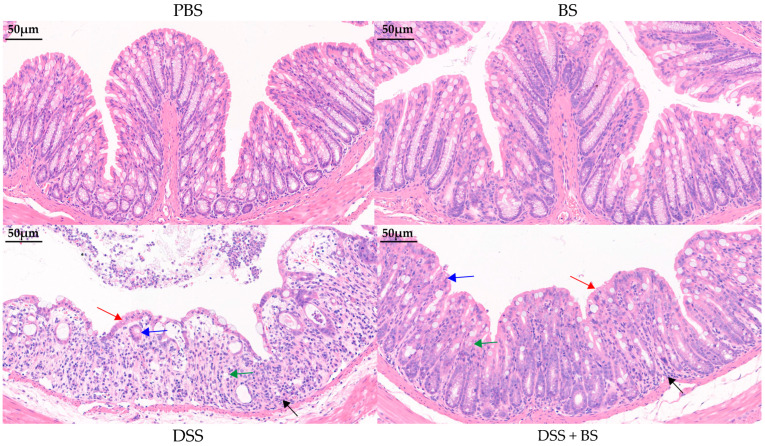
HE staining of mouse colon tissue sections. The red arrow indicates intestinal mucosal damage, the black arrow indicates inflammatory cell infiltration, the blue arrow indicates crypt loss, and the green arrow indicates reduced goblet cell numbers (bar = 50 μm).

By further observing the pathological sections of colonic histology (Figure 16), it can be noted that the intestinal villi structure remains intact in both the PBS group and the BS group. In contrast, the intestinal villi in the DSS group exhibit severe structural damage, with extensive loss of mucosal epithelium, a marked reduction in the number of goblet cells and crypts, and significant infiltration of inflammatory cells in the submucosa. In the DSS + BS group, where *Bacillus subtilis* was administered, the extent of mucosal epithelial damage and inflammatory cell infiltration was notably reduced compared to the DSS group, and the number of goblet cells and crypts was comparatively higher.

#### 3.5.3. The Effect of *Bacillus subtilis* on Tight Junction Proteins in Colonic Tissue

**Figure 17:**
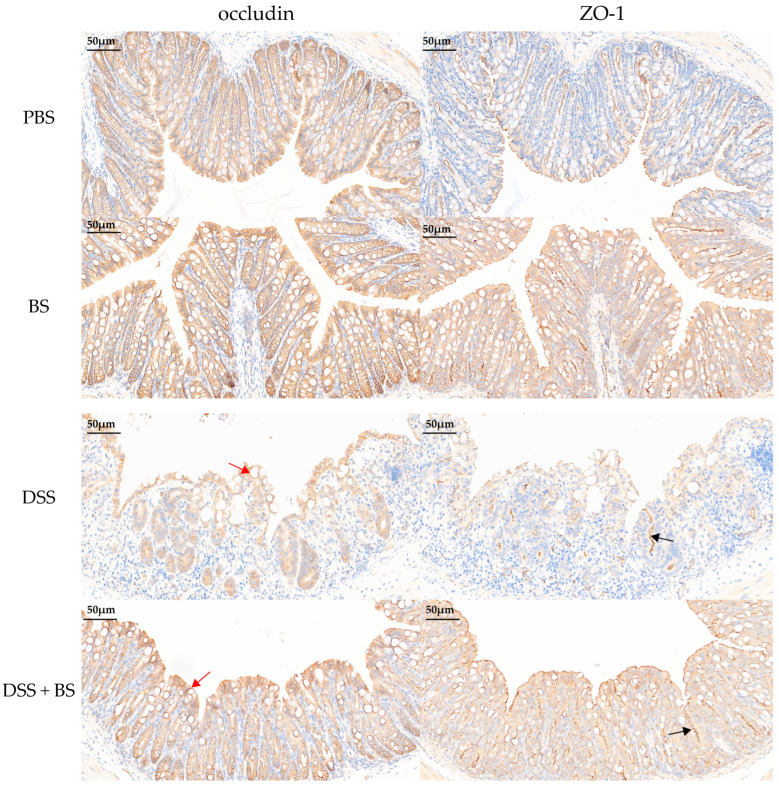
IHC images of the mice intestine. The red arrow indicates a decrease in occludin protein expression, while the black arrow indicates a decrease in ZO-1 protein expression (bar = 50 μm).

**Figure 18:**
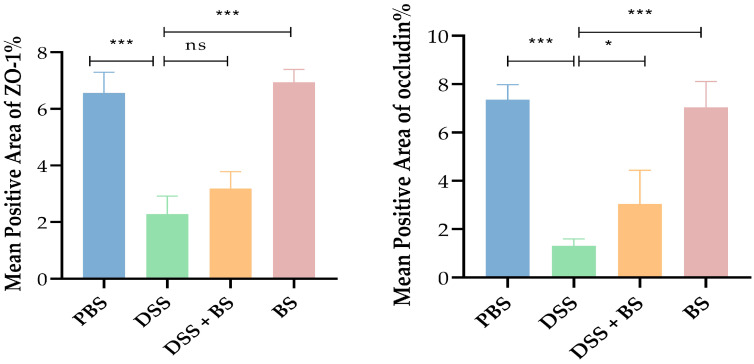
Analysis results of IHC slide average optical density. The results are presented as mean ± SEM. The difference is not statistically significant (ns). * *p* < 0.05 indicates a significant difference, and *** *p* < 0.001 indicates an extremely significant difference (*n* = 6).

In models of DSS-induced IBD, the expression levels of these tight junction proteins can serve as indicators of intestinal damage. The effects of DSS-induced IBD and the administration of *Bacillus subtilis* on colonic tight junction protein (ZO-1 and Occludin) expression were assessed using IHC and semi-quantitative analysis (Figure 17 and Figure 18), with brownish-yellow staining indicating positive reactivity. The results demonstrated that, compared to the PBS control group, mice treated with DSS for 7 days exhibited a significant reduction in the expression of colonic tight junction proteins. However, in the group treated with *Bacillus subtilis*, the expression of these proteins was notably higher compared to the DSS-only group.

#### 3.5.4. The Effect of *Bacillus subtilis* on the Expression of Inflammatory Factors

**Figure 19:**
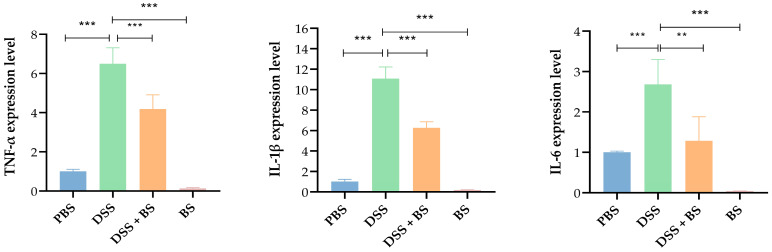
The expression levels of inflammatory factors. The results are presented as mean ± SEM, ** *p* < 0.01 indicates a significant difference, and *** *p* < 0.001 indicates an extremely significant difference (*n* = 4).

Quantitative PCR analysis revealed that, compared with the PBS control group, the DSS group exhibited significantly increased expression levels of pro-inflammatory cytokines such as TNF-α, IL-1β, and IL-6. In contrast, the DSS + BS group showed reduced expression of these cytokines compared to the DSS group. Notably, mice in the *Bacillus subtilis* group exhibited lower expression levels of pro-inflammatory factors than those in PBS group (Figure 19), suggesting that *Bacillus subtilis* may exert anti-inflammatory effects by downregulating the expression of inflammatory mediators, thereby mitigating the inflammatory response.

## 4. Discussion

*Bacillus subtilis* can be utilized as a feed additive in livestock and poultry farming, offering benefits such as enhancing feed nutritional value, improving feed conversion efficiency, and promoting intestinal health in animals [23]. For *Bacillus subtilis* to function effectively in animal feed, it must remain viable in the gastrointestinal tract. Therefore, it should exhibit resistance to acidic and alkaline conditions, bile salts, and high temperatures encountered during feed processing [24]. Research indicates that the gastric pH in animals typically ranges from 2 to 3, while the intestinal pH ranges from 5 to 7, with bile salt concentrations generally between 0.03% and 0.30% [25]. In the present study, a strain of *Bacillus subtilis* which was isolated from goats in Tongcheng, Hubei Province, was evaluated for its tolerance to acidic, alkaline, and high-temperature environments. The results demonstrated that after incubation at pH 2.0–4.0 for 3 h, the survival rate of the *Bacillus subtilis* ranged from 27% to 57%. When exposed to pH 8.0–10.0 for the same duration, the survival rate was between 28% and 51%. Compared with another isolated strain of *Bacillus subtilis*, the results demonstrated that the isolated strain in the present study exhibited superior acid tolerance, enhancing its viability during gastrointestinal transit [26]. At a bile salt concentration of 0.1%, the survival rate was 68%, which decreased to 43% at 0.3%, and the strain still survived at a concentration of 0.5%. In simulated gastrointestinal fluid tests, the survival rate decreased from 48% to 35% after 3 h of exposure to artificial gastric juice, and remained at 17.7% after 3 h in artificial intestinal fluid. In the heat resistance test, the strain remained viable at 80 °C, with a survival rate of 10%. These findings indicated that the isolated strain of *Bacillus subtilis* possesses a certain degree of environmental stress tolerance.

To further analyze the genomic bioinformatics characteristics of the isolated strain of *Bacillus subtilis*, as well as its probiotic properties, this study performed whole-genome sequencing and functional analysis based on experimental data, including assessments of acid and alkali resistance, salt tolerance, and self-aggregation capacity, in order to elucidate its genomic features and predict potential functions [27]. The results of the self-aggregation capacity assay demonstrated that the isolated strain exhibits a higher aggregation rate compared to another *Bacillus subtilis* isolate [28].

Studies have shown that carbohydrate-active enzymes are encoded by numerous genes within the microbial genome [28] and are associated with the host’s nutrient absorption processes. Among these enzymes, glycoside hydrolases catalyze the hydrolysis of glycosidic bonds and play a crucial role in the breakdown and synthesis of sugars and sugar conjugates in biological systems [29]. Glycosyltransferases, on the other hand, are involved in the formation of glycosidic bonds and the synthesis of various sugar structures, and exhibit potential immunomodulatory properties [30]. Genomic sequencing analysis has revealed that the genome of the isolated strain of *Bacillus* contains genes encoding both glycoside hydrolases and glycosyltransferases, indicating that the isolated strain possesses the capability to synthesize carbohydrates and metabolize sugars effectively.

Vitamins are trace organic compounds that cannot be synthesized by the human or animal body and must therefore be obtained through dietary sources to maintain normal physiological functions. They play a crucial role in the growth and development of animals. Research indicates that vitamins function as potent signaling molecules through interactions with various nuclear receptors and cellular signaling pathways, exerting inhibitory effects on inflammatory factors [31]. Sequencing data reveal that the isolated strain of *Bacillus subtilis* contains genes involved in the synthesis of cofactors and vitamin metabolism. As a feed additive, *Bacillus subtilis* must survive within the gastrointestinal tract of animals and establish intestinal adhesion and colonization. Gene Ontology database analyses indicated that the isolated strain possesses genes associated with catalytic activity and adhesion at the molecular function level.

Previous studies have demonstrated that probiotics generally lack genetic elements associated with transmissible antibiotic resistance, making antibiotic susceptibility an essential criterion for evaluating their safety during screening processes [32]. The isolated strain of *Bacillus subtilis* harbors multiple antibiotic resistance genes, with the highest prevalence being peptide antibiotic resistance genes. However, sequencing results indicate that the isolated strain does not possess plasmids, suggesting a low likelihood of horizontal gene transfer of these resistance genes to other intestinal pathogens. Nevertheless, the potential risk of resistance gene transfer cannot be entirely ruled out at the genomic level [33].

The supplementation of prebiotics and probiotics may be associated with reduced adipose tissue inflammation, decreased systemic inflammation, and a lower risk of chronic diseases [34]. *Bacillus subtilis* had been shown to inhibit the activation of the NF-κB inflammatory pathway in the jejunum and ileum of piglets, reduce levels of pro-inflammatory cytokines in both blood and the small intestine, enhance the expression of intestinal tight junction proteins, and improve the composition of the colonic microbiota [35,36]. Intestinal epithelial tight junction proteins play a crucial role in maintaining the integrity of the intestinal mucosal barrier [37]. The findings in the present study indicated that administration of *Bacillus subtilis* can partially mitigate DSS-induced damage to intestinal tight junctions and help preserve the integrity of the intestinal barrier in mice.

IBD is a multifactorial condition characterized by chronic intestinal inflammation, such as ulcerative colitis. Common clinical manifestations include diarrhea with blood, weight loss, and fatigue. Currently, pharmacological treatments for IBD may lead to significant adverse effects and pose potential risks to overall health. In recent years, probiotics have increasingly been explored for their potential in the prevention and treatment of inflammatory disorders. A recent study demonstrated that *Bacillus subtilis* capable of producing exopolysaccharides can mitigate inflammation by modulating the innate immune response and protect mice against Citrobacter rodentium-induced acute colitis [38]. The development of industrial products from the isolated strain can be enhanced through the application of nanotechnology, which enables the fabrication of nanoparticles to improve its efficacy [39,40].

Inflammatory mediators play a crucial role in the pathogenesis of IBD and serve as key biomarkers for assessing the severity of inflammation. Among these mediators, cytokines such as TNF-α and members of the interleukin (IL) family, including IL-1β and IL-6, are secreted by macrophages, dendritic cells, and epithelial cells, contributing to the initiation and perpetuation of inflammatory responses [41]. In models of DSS-induced acute colitis, macrophages, neutrophils, and eosinophils accumulate in the intestinal tissue, leading to inflammatory infiltration [42]. Research has demonstrated that elevated levels of pro-inflammatory cytokines can compromise intestinal barrier function by disrupting the integrity and tight junctions of the epithelial cell layer [43]. The present study aimed to investigate whether the *Bacillus subtilis* isolated could alleviate colitis in mice. The results demonstrated that in the DSS-induced mouse colitis model, the intestinal villus epithelial cells were severely damaged in the DSS group, accompanied by a reduction in the number of crypts and goblet cells, as well as decreased expression of intestinal tight junction proteins. Additionally, the expression levels of pro-inflammatory cytokines, including TNF-α, IL-1β, and IL-6, were significantly elevated. In terms of anti-inflammatory cytokine IL-10, the *Bacillus subtilis* group exhibited higher expression level than those in PBS group, DSS + BS group, and DSS group. These findings indicated that the colitis model in mice was successfully set up. In contrast, the DSS + BS group, which received treatment with the *Bacillus subtilis* isolated strain suspension, exhibited milder pathological changes than the DSS group. Specifically, there was less damage to intestinal villus epithelial cells and reduced infiltration of inflammatory cells. Moreover, the expression levels of intestinal tight junction proteins were higher, while the levels of TNF-α, IL-1β, and IL-6 were lower. These results suggested that the isolated strain of *Bacillus subtilis* can alleviate the symptoms of colitis in mice.

With the development of large-scale goat farming, intestinal diseases in goats have become increasingly prevalent [44]. The extensive use of antibiotics not only contributes to the emergence of antimicrobial resistance but also results in antibiotic residues in mutton, thereby posing a significant risk to food safety [45]. Investigating the use of probiotics for the prevention and treatment of intestinal diseases in goats represents a safe and effective strategy. In the present study, a *Bacillus subtilis* strain was isolated from goats. This is a local elite strain that exhibits superior performance in the unique intestinal environment of goat. Owing to inherent host adaptability, specialized metabolic functions, and an improved safety profile, the isolated strain is anticipated to confer more substantial and consistent benefits in terms of enhancing ruminant health, feed efficiency, and production performance relative to conventional probiotics.

## 5. Conclusions

In summary, this study first characterized the genome and genetic properties of *Bacillus subtilis* isolated from goats. Results indicated that the genome of *Bacillus subtilis* contained genes related to cellular processes and metabolic activities. Moreover, we performed functional prediction on the genome and found that some genes were involved in amino acid transport and metabolism, transcription, carbohydrate transport and metabolism, inorganic ion transport and metabolism, etc., which further confirmed that *Bacillus subtilis* isolated from goats has good probiotic properties. This study revealed the beneficial properties of *Bacillus subtilis* from the genomic perspective, which contributed to providing a theoretical basis for the developing probiotic products of *Bacillus subtilis*. However, further investigations are required to demonstrate the probiotic properties of the isolated strain in goats in natural conditions.

## Figures and Tables

**Table 1:** DAI Specific Scoring Criteria.

Weight Loss %	Blood in Stool	Form of Stool	Rating Value/Score
<1	No	Normal	0
≥1–5	No	Normal	1
≥5–10	Vision particle bleeding	Slimy	2
≥10–15	Crimson bloody stool, blood around the anus	Slimy	3
≥15	Massive bleeding	Diarrhea	4

**Table 2:** The primer sequences in the present study.

Gene Name	Forward Primer (5′ → 3′)	Reverse Primer (5′ → 3′)	Reference Gene
β-actin	CGGTCAGGATCTTCATGAGGTAG	CATGTTTGAGACCTTCAACACCC	XM_051205325.1
TNF-α	TTTCCAGATTCTTCCCTGAGGTG	AAAGAGGAGGCAACAAGGTAGAG	M20155.1
IL-1β	GTGGTAAATGAAACCTGTGTGGG	CTGTTCTTTGAAGTTGACGGACC	X04964.1
IL-6	CGATAGTCAATTCCAGAAACCGC	CTCCACTCAAAACCAGCAAAGAG	M20572.1

**Table 3:** Quality control information for the isolated *Bacillus subtilis*.

Sample ID	Number	Bases	Largest Length	N50 Length	N90 Length
isolated *Bacillus subtilis*	603,722	25,415,378,888	69,930	8460	1830

**Table 4:** The prediction results for coding genes.

Sample ID	Gene Num	Gene Total Length	Gene Average Length
Isolated *Bacillus subtilis*	4276	366,822	857

**Table 5:** The prediction results for non-coding genes.

Sample ID	ncRNA Type	Number	Average Length (bp)	Total Length (bp)
Isolated *Bacillus subtilis*	tRNA	86	77	6643
Isolated *Bacillus subtilis*	5S rRN5A	10	115	1150
Isolated *Bacillus subtilis*	16S rRNA	10	1538	15,380
Isolated *Bacillus subtilis*	23S rRNA	10	2926	29,260

## Data Availability

All sequences in this study are available from the NCBI BioProject number PRJNA 1307218. All data generated or analyzed during the present study have been submitted with this manuscript.

## Abbreviations

The following abbreviations are used in this manuscript:

BW	body weight
CARD	Comprehensive Antibiotic Resistance Database
COG	Clusters of Orthologous Groups
DAI	Disease Activity Index
DSS	dextran sulfate sodium
HE	hematoxylin and eosin
IBD	inflammatory bowel disease
IHC	immunohistochemical
KEGG	Kyoto Encyclopedia of Genes and Genomes
qRT-PCR	Quantitative reverse transcription polymerase chain reaction
TSA	tryptone soy agar
TSB	tryptone soy broth
